# Real-World Accuracy of Wearable Activity Trackers for Detecting Medical Conditions: Systematic Review and Meta-Analysis

**DOI:** 10.2196/56972

**Published:** 2024-08-30

**Authors:** Ben Singh, Sebastien Chastin, Aaron Miatke, Rachel Curtis, Dorothea Dumuid, Jacinta Brinsley, Ty Ferguson, Kimberley Szeto, Catherine Simpson, Emily Eglitis, Iris Willems, Carol Maher

**Affiliations:** 1 Allied Health & Human Performance University of South Australia Adelaide Australia; 2 Department of Rehabilitation Sciences and Physiotherapy Ghent University Ghent Belgium

**Keywords:** wearable activity trackers, disease detection, atrial fibrillation, COVID-19 diagnosis, meta-analysis, wearables, wearable tracker, tracker, detection, monitoring, physiological, diagnostic tool, tool, tools, Fitbit, atrial, COVID-19, wearable

## Abstract

**Background:**

Wearable activity trackers, including fitness bands and smartwatches, offer the potential for disease detection by monitoring physiological parameters. However, their accuracy as specific disease diagnostic tools remains uncertain.

**Objective:**

This systematic review and meta-analysis aims to evaluate whether wearable activity trackers can be used to detect disease and medical events.

**Methods:**

Ten electronic databases were searched for studies published from inception to April 1, 2023. Studies were eligible if they used a wearable activity tracker to diagnose or detect a medical condition or event (eg, falls) in free-living conditions in adults. Meta-analyses were performed to assess the overall area under the curve (%), accuracy (%), sensitivity (%), specificity (%), and positive predictive value (%). Subgroup analyses were performed to assess device type (Fitbit, Oura ring, and mixed). The risk of bias was assessed using the Joanna Briggs Institute Critical Appraisal Checklist for Diagnostic Test Accuracy Studies.

**Results:**

A total of 28 studies were included, involving a total of 1,226,801 participants (age range 28.6-78.3). In total, 16 (57%) studies used wearables for diagnosis of COVID-19, 5 (18%) studies for atrial fibrillation, 3 (11%) studies for arrhythmia or abnormal pulse, 3 (11%) studies for falls, and 1 (4%) study for viral symptoms. The devices used were Fitbit (n=6), Apple watch (n=6), Oura ring (n=3), a combination of devices (n=7), Empatica E4 (n=1), Dynaport MoveMonitor (n=2), Samsung Galaxy Watch (n=1), and other or not specified (n=2). For COVID-19 detection, meta-analyses showed a pooled area under the curve of 80.2% (95% CI 71.0%-89.3%), an accuracy of 87.5% (95% CI 81.6%-93.5%), a sensitivity of 79.5% (95% CI 67.7%-91.3%), and specificity of 76.8% (95% CI 69.4%-84.1%). For atrial fibrillation detection, pooled positive predictive value was 87.4% (95% CI 75.7%-99.1%), sensitivity was 94.2% (95% CI 88.7%-99.7%), and specificity was 95.3% (95% CI 91.8%-98.8%). For fall detection, pooled sensitivity was 81.9% (95% CI 75.1%-88.1%) and specificity was 62.5% (95% CI 14.4%-100%).

**Conclusions:**

Wearable activity trackers show promise in disease detection, with notable accuracy in identifying atrial fibrillation and COVID-19. While these findings are encouraging, further research and improvements are required to enhance their diagnostic precision and applicability.

**Trial Registration:**

Prospero CRD42023407867; https://www.crd.york.ac.uk/prospero/display_record.php?RecordID=407867

## Introduction

As health care budgets around the world continue to soar, the need for cost-effective interventions that both reduce health care costs and improve patient outcomes has never been more urgent [1]. Early detection of medical conditions offers a pathway to achieve these goals, enabling prompt intervention during acute medical events or even pre-emptive action before such events occur [2]. Wearable activity monitors are emerging as a potential tool in this evolving landscape.

In recent years, wearable activity trackers have become ubiquitous tools, widely adopted for tracking and enhancing physical activity and other lifestyle behaviors, helping to mitigate the risk of chronic diseases [3]. These devices measure a plethora of activity metrics such as steps taken, distance covered, energy expenditure, physical activity intensities, and sleep patterns [4]. The scientific literature has witnessed a surge in original studies and systematic reviews and meta-analyses, focused on determining the reliability and validity of activity trackers for measuring activity levels [5,6] and their effectiveness in intervening in daily activity patterns and downstream health outcomes [7-12]. These studies have shown that interventions using consumer-based wearable activity trackers can increase physical activity participation and lead to significant improvements in health outcomes, across a range of populations [7-12]. As wearable technology has progressed, wearable activity trackers offer increasing potential to move beyond activity metrics and aid in the early identification of diseases and other medical events.

Rapid technological advancements have significantly extended the capabilities of contemporary consumer-grade wearable activity trackers such as Fitbits and Apple Watches [13]. Modern wearables incorporate sophisticated sensors capable of monitoring a wide array of physiological parameters beyond just movement including heart rate, blood oxygen levels, sleep quality, and stress markers [14]. While this expanded functionality holds promise for disease detection and monitoring, the evidence supporting the use of consumer wearables for such applications remains limited. For example, the systematic review by Alban-Cadena et al [15] evaluated wearable sensors for monitoring Parkinson disease–related gait impairments and symptoms such as tremors, bradykinesia, and dyskinesia. However, most included studies were very small (10-20 participants) and were conducted in controlled laboratory environments using specialized setups such as multi-sensor accelerometer arrays worn on the ankles and spine. While offering the potential for home-based rehabilitation, the generalizability of these findings to widely adopted, consumer-oriented wearable trackers designed for real-world, free-living conditions is unclear.

Other recent systematic reviews have evaluated the accuracy of wearable tracking devices for detecting specific health conditions such as arrhythmias [16], cardiovascular disease [17,18], and COVID-19 [19]. However, these reviews have notable limitations. Most included studies were conducted in controlled laboratory settings, limiting the generalizability of their findings to real-world, free-living conditions [16,17,19]. Additionally, these reviews focused narrowly on individual clinical outcomes, preventing comparisons of wearables’ detection accuracy across different medical conditions and events. For example, the narrative syntheses highlighted wearables’ potential as complementary tools for detecting cardiovascular conditions such as arrhythmias, atrial fibrillation, myocardial infarction, and heart failure [16,17]. The meta-analysis of Lee et al [18] of 26 studies found wearable devices had a pooled sensitivity of 94.80% and specificity of 96.96% for atrial fibrillation detection. In contrast, Cheong et al [19] reported lower diagnostic accuracy for COVID-19 detection, with area under the curve (AUC) values ranging from 75% to 94.4% and sensitivity and specificity ranging from 36.5% to 100% and 73% to 95.3%, respectively. Notably, all but one review [18] used narrative synthesis approaches [16,17,19], limiting their ability to quantify detection accuracy, and preventing readers from comparing detection accuracy across conditions reported in the respective reviews.

As wearable technology rapidly evolves, with frequent introductions of new and more advanced devices, the scientific evidence base for disease detection is growing, encompassing a wider range of medical conditions and events. Consequently, there is now sufficient data to warrant a comprehensive systematic review with meta-analyses, allowing quantitative comparisons of wearables’ detection accuracy across various conditions in real-world settings.

Our systematic review and meta-analysis aim to fill this crucial gap by comprehensively assessing the reliability and accuracy of consumer-grade wearable activity trackers for detecting and monitoring a wide range of medical conditions and events in free-living, real-world settings. Unlike previous reviews that relied on narrative synthesis approaches, our quantitative meta-analyses will allow for robust comparisons of wearables’ diagnostic performance across diverse conditions and events. By rigorously evaluating evidence from studies conducted in real-world contexts, our review will provide evidence to guide the responsible and effective implementation of wearable technology for early detection and continuous health monitoring by researchers, health care providers, policy makers, technology companies, and other stakeholders. As consumer adoption of wearables continues to rise rapidly worldwide, our comprehensive synthesis will assist in harnessing their potential while mitigating risks and ensuring appropriate use.

## Methods

### Protocol and Registration

The protocol for this systematic review was prospectively registered on PROSPERO (ID CRD42023407867) and this paper is reported according to PRISMA (Preferred Reporting Items for Systematic Reviews and Meta-Analyses) [20] guidelines.

### Selection Criteria and Search Strategy

The inclusion criteria are summarized in Table S1 in Multimedia Appendix 1. The inclusion criteria were developed using the population, exposure, outcomes, and study type criteria as follows: adult population (aged 18 years or older) in free-living conditions, that have not been recruited based on a specific health condition or diagnosis; use of a wearable activity tracker (eg, Fitbit, Apple Watch, or a research-grade accelerometer) for the detection of any disease or medical event (eg, atrial fibrillation, the onset of infectious disease, and falls). To be eligible, the wearable activity tracker had to be able to detect movement behavior (ie, include an accelerometer), but could also include other types of sensors (eg, light sensor and temperature sensor). The wearable activity tracker had to consist of a single device worn on a single body location (eg, on the wrist or chest, not across both); studies needed to assess the actual diagnosis of a medical condition or occurrence of events that had clinical relevance (eg, falls). Eligible studies are needed to report an outcome related to diagnostic accuracy, such as specificity or sensitivity of the device for early detection of disease or medical events. Examples could include but were not limited to, providing effect estimates of overall diagnostic accuracy (%), sensitivity (%), and specificity (%) with 95% CIs; and validation studies conducted under free-living conditions that were reported in a peer-reviewed journal study were included. This included secondary analyses conducted within the context of observational studies, experimental studies, or quasi-experimental studies. Both consumer-initiated studies, where existing consumers who had purchased their own wearables were invited to join a study, and researcher-initiated studies, where researchers recruited participants and provided them with wearables, were included, as they represent 2 complementary real-world contexts in which wearable devices are often implemented for disease detection and monitoring. Studies were included only if they evaluated wearable devices provided by health care providers or researchers as part of a formal monitoring program, and the detection of a specific clinical event or disease was a prespecified outcome measure of the study. Studies examining consumer-driven self-tracking with personal wearables outside of a health care context were excluded. The following were also excluded: studies involving children or adolescents, studies examining symptoms within people known to have a specific disease, wearable devices that cannot track activity levels (eg, continuous glucose monitors), studies evaluating an array of wearable sensors worn at multiple body locations (eg, watch plus skin patch) or pedometers, studies measuring the association between an exposure and an outcome (eg, using odds ratios, relative risk, and hazard ratios), lab- or hospital-based studies, and conference abstracts or dissertations.

Ten databases were searched (CINAHL, Cochrane Library, Embase via OVID, MEDLINE via OVID, Emcare via OVID, JMIR Publications, ProQuest Central, ProQuest Nursing and Allied Health Source, PsycINFO, and Scopus) using subject heading, keyword, and MeSH (Medical Subject Headings) term searches for terms related to “wearable device” and “detection” (see Table S2 in Multimedia Appendix 1 for the full search strategy). We intentionally used broad search terms to ensure a comprehensive capture of the evidence base, including all types of medical conditions and events, without restricting our search to predefined diagnostic or event outcomes. Database searches were limited to peer-reviewed journal studies published in English from inception to April 1, 2023.

### Data Management and Extraction

Search results were imported into ASReview (version 2.0; ASReview Community), an open-source software artificial intelligence tool designed for screening studies for systematic reviews. Title or abstract screening was conducted in ASReview by paired independent reviewers (BS and DD, RC, TF, JB, IW, KS, CS, AM, or EE). The software uses an active learning algorithm that iteratively selects the most relevant studies for inclusion based on the initial judgments made by the research team. The screening was stopped when 100 consecutive nonrelevant studies were screened. Following title or abstract screening, results were then imported to EndNote X9 (Clarivate) where duplicates were removed and then exported into Covidence (Veritas Health Innovation) for full-text screening, data extraction, and risk of bias scoring which was completed in duplicate by paired independent reviewers (BS and DD, RC, TF, JB, IW, KS, CS, AM, or EE), with disagreements resolved by discussion.

Data were extracted in duplicate by paired independent reviewers (BS and DD, RC, TF, JB, IW, KS, CS, AM, or EE) using a standardized extraction form in Covidence. The risk of bias in the included reviews was assessed by 2 independent reviewers in duplicate using the Joanna Briggs Institute (JBI) Critical Appraisal Checklist for Diagnostic Test Accuracy Studies. Studies were rated out of nine for the following items: (1) enrollment of consecutive or random sample, (2) the avoidance of a case-control design, (3) inappropriate exclusions, (4) the interpretation of index test results, (5) the prespecification of thresholds, (6) reference standard classification, (7) the interpretation of reference standard, (8) timing of tests, and (9) analysis.

### Data Synthesis and Analysis

For each meta-analysis, data were combined at the study level. Separate meta-analyses were performed for (1) COVID-19 detection, (2) atrial fibrillation or arrhythmia detection, and (3) fall detection. Outcomes of interest were analyzed and data were pooled using sensitivity (%), specificity (%), AUC (%), accuracy (%), and positive predictive value (PPV), with 95% CIs as the effects measures. Sensitivity (%) denotes the percentage of individuals with the disease or condition correctly identified by the test, while specificity (%) represents the percentage of those without the disease or condition correctly identified as negative. The AUC (%) quantifies the test’s overall diagnostic accuracy, ranging from 0% to 100%, with higher values indicating better performance. Accuracy (%) reflects the proportion of all tests accurately classified, and PPV (%) indicates the likelihood that a positive test result correlates with the disease or condition being tested for. If 95% CIs were not reported in a study, they were calculated based on available data, using recommended formulas [21]. Publication bias was evaluated using funnel plots of effect sizes and standard errors and evaluating for asymmetries or missing sections within the plot, for meta-analyses that involved more than 10 studies. The Cochran Q test was used to assess statistical heterogeneity and the *I*^2^ statistic was used to quantify the proportion of the overall outcome attributed to variability. The following cut-off values for the *I*^2^ statistic were used: 0% to 29%=no heterogeneity; 30% to 49%=moderate heterogeneity; 50% to 74%=substantial heterogeneity; and 75% to 100%=considerable heterogeneity [22]. Subgroup analyses were undertaken to evaluate device type (Fitbit, Apple watch, Oura ring, and others) for outcomes that had at least 2 studies in each subgroup. Sensitivity analyses for the meta-analysis were performed by removing the study with the lowest sensitivity, specificity, AUC, accuracy, or PPV. All meta-analyses were performed using Stata/MP (version 16; StataCorp).

The overall level of evidence was graded using the Oxford Centre for Evidence-Based Medicine 2011 Levels of Evidence, as follows: grade A: consistent level 1 studies (ie, individual randomized controlled trials); B: consistent level 2 (ie, individual cohort studies) or 3 studies (ie, individual case-control studies) or extrapolations from level 1 studies; C: level 4 studies (ie, case series) or extrapolations from level 2 or 3 studies; or D: level 5 (ie, expert opinion without explicit critical appraisal) evidence or inconsistent or inconclusive studies of any level [23]. Each outcome of interest was assigned a “Grade of Recommendation” based on meeting these criteria.

### Deviations From the Registered Protocol

We planned to use the Effective Public Health Practice Project Quality Assessment Tool to assess study quality and risk of bias. However, during data extraction and quality assessment, we opted to use the JBI Critical Appraisal Checklist for Diagnostic Test for Accuracy Studies, as this instrument was more relevant to the included studies. Further, we were unable to conduct subgroup analyses for the type of wearable for atrial fibrillation and fall detection, due to an insufficient number of studies.

## Results

### Overview

**Figure 1:**
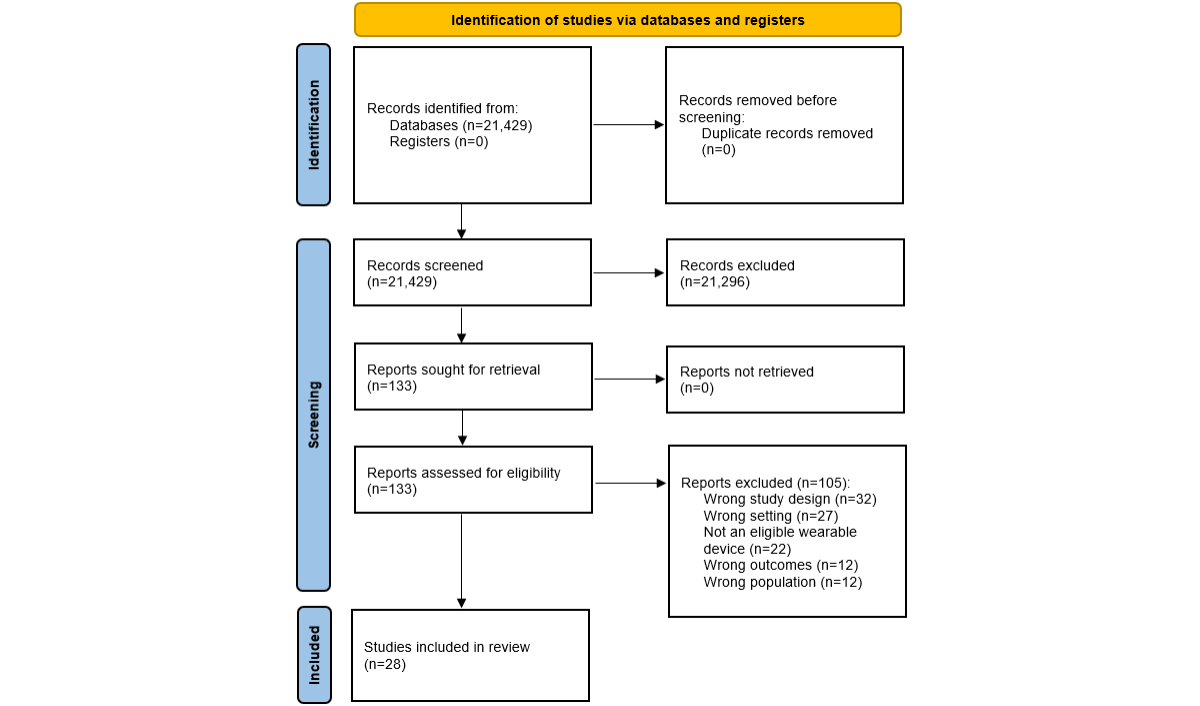
PRISMA (Preferred Reporting Items for Systematic Reviews and Meta-Analyses) flow diagram.

Of the 21,429 records identified following the database search, 28 were eligible (see Figure 1 for PRISMA flowchart including reasons for exclusions; see Table S3 in Multimedia Appendix 1 for a complete list of full texts that were excluded during the final stage of screening, with reasons). An overview of all included study’s characteristics is shown in Table S4 in Multimedia Appendix 1. There was a total of 1,226,801 participants (median sample size 264, IQR 96-8338; range 29-455,699). Median participant age was 47.3 (IQR 36.6-66), between 28.6 and 78.3, years and 21 (75%) studies involved female and male participants (gender was not reported in 7 (25%) studies). A total of 16 (57%) studies evaluated COVID-19, 5 (18%) studies evaluated atrial fibrillation, 3 (11%) studies assessed a broad range of cardiac arrhythmias, 3 (11%) studies assessed falls, and 1 (3.6%) study assessed viral symptoms. The devices used in the studies were Fitbit (n=6), Apple Watch (n=6), Oura ring (n=3), a combination of various devices (ie, studies that used a combination of the Apple Watch, Fitbit, Garmin, and other devices; n=7), Empatica E4 (n=1), Dynaport MoveMonitor (n=2), Samsung Galaxy Watch (n=1), and other or not specified (n=2). The median score for the JBI Critical Appraisal Checklist for Diagnostic Test Accuracy Studies was 6 (IQR 5-7; range 1-9) out of 9 (Table S5 in Multimedia Appendix 1).

There was sufficient data in the included studies to conduct meta-analyses for the following clinimetrics: (1) COVID-19 detection (accuracy, %; sensitivity, %; AUC, %; and specificity, %), (2) atrial fibrillation detection (PPV, %; sensitivity, %; and specificity, %), and (3) falls detection (sensitivity, %; and specificity, %).

### Meta-Analysis Results

#### COVID-19 Detection

**Figure 2:**
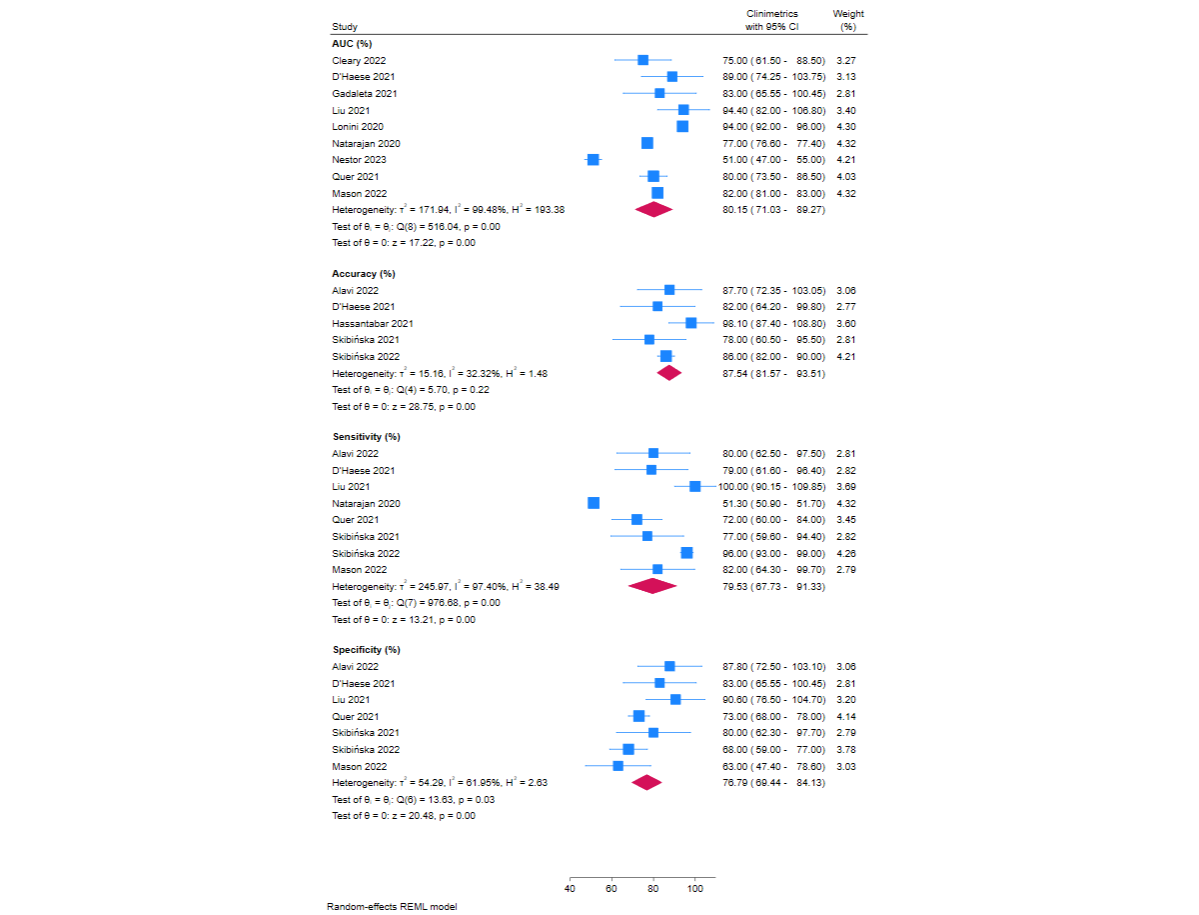
Meta-analysis of accuracy, sensitivity, AUC, and specificity of wearable activity trackers for detection of COVID-19. AUC: area under the curve.

Meta-analysis results of AUC, accuracy, sensitivity, and specificity for COVID-19 detection are shown in Figure 2. Meta-analyses of 9 studies showed a pooled AUC of 80.15% (95% CI 71.03%-89.27%) and 5 studies had a pooled accuracy of 87.54% (95% CI 81.57%-93.51%). Pooled sensitivity from 8 studies was 79.53% (95% CI 67.73%-91.33%), and 7 studies showed a pooled specificity of 76.79% (95% CI 69.44%-84.13%).

**Figure 3:**
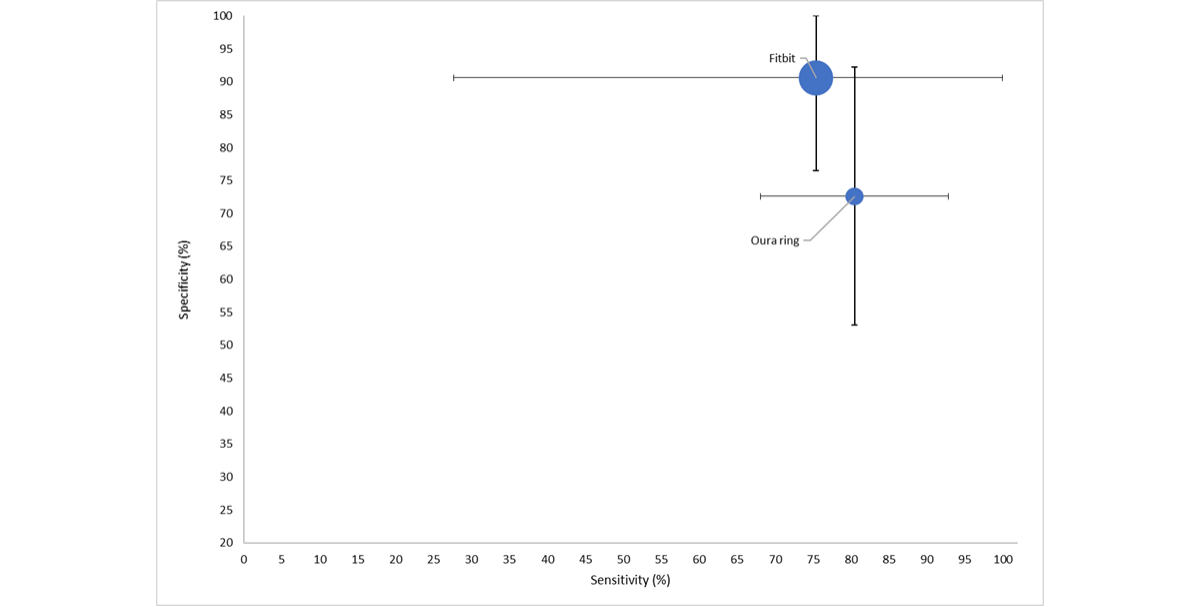
Overview of sensitivity and specificity for the different devices for COVID-19 detection.

Subgroup analysis for device type for sensitivity and specificity are shown in Figures S6 and S7 in Multimedia Appendix 1, respectively. A summary of sensitivity and specificity for the different devices is shown in Figure 3. Overall, the Fitbit had a sensitivity and specificity of 75.39% and 90.60%, respectively, the Oura ring had a sensitivity and specificity of 80.47% and 72.60%, respectively, and combined devices had a sensitivity and specificity of 82.69% and 74.62%, respectively.

The results of sensitivity analyses are shown in Figure S3 in Multimedia Appendix 1. Following the removal of the worst-performing study, AUC was 84.10%, accuracy was 88.65%, sensitivity was 85.62%, and specificity was 78.57%.

Grade of recommendation: (B) consistent level 2 studies supporting the use of wearable activity trackers for the detection of COVID-19.

#### Atrial Fibrillation Detection

**Figure 4:**
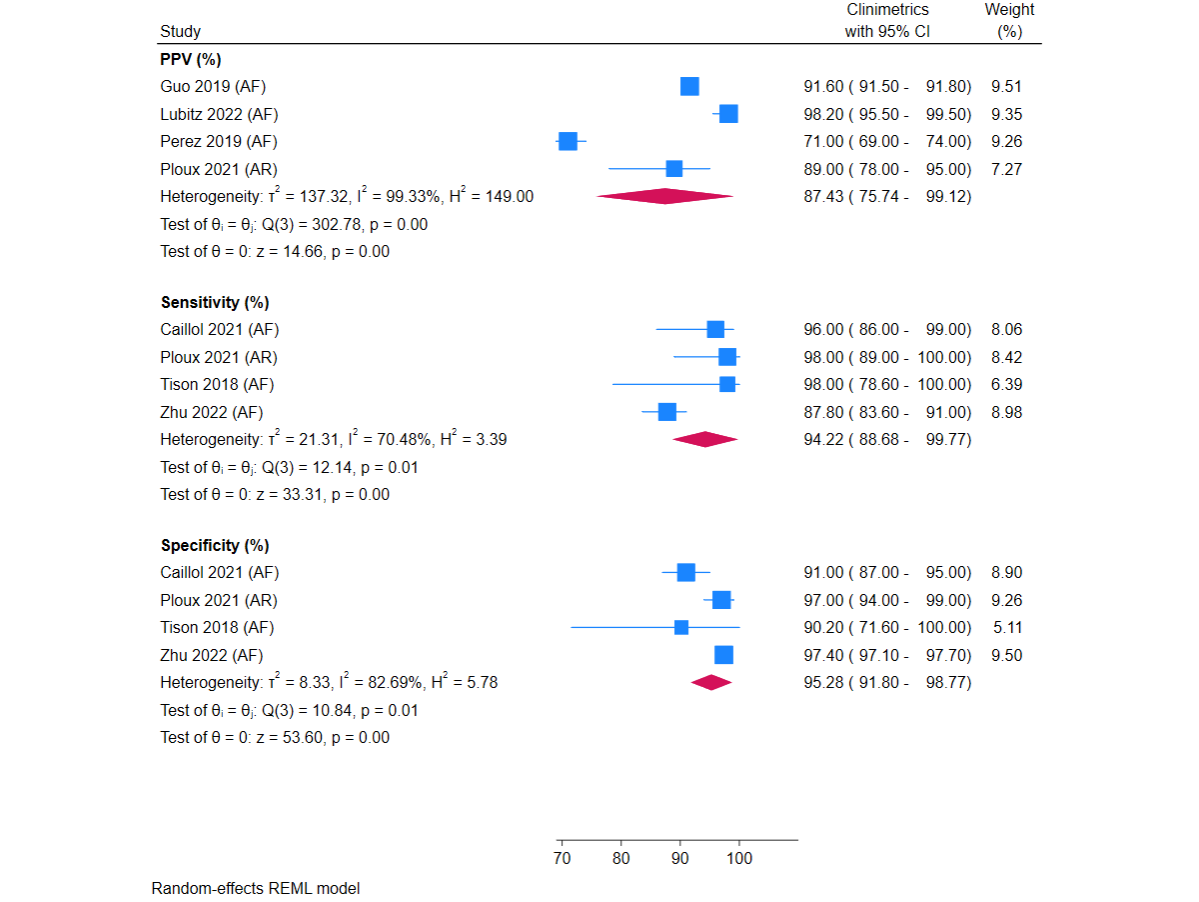
Meta-analysis of PPV, sensitivity, and specificity of wearable activity trackers for detection of AF and AR. AF: atrial fibrillation; AR: arrhythmia; PPV: positive predictive value.

Pooled analyses of PPV, sensitivity, and specificity for atrial fibrillation detection are shown in Figure 4. Meta-analysis of 4 studies showed a combined PPV of 87.43% (95% CI 75.74%-99.12%). Pooled sensitivity was 94.22% (95% CI 88.68%-99.77%; 4 studies) and pooled specificity was 95.28% (95% CI 91.80%-98.77%; 4 studies).

The results of sensitivity analyses are shown in Figure S4 in Multimedia Appendix 1. Following the removal of the worst-performing study, PPV was 93.64%, sensitivity was 97.28%, and specificity was 95.55%.

Grade of recommendation: (B) consistent level 2 studies supporting the use of wearable activity trackers for the detection of atrial fibrillation.

#### Falls Detection

**Figure 5:**
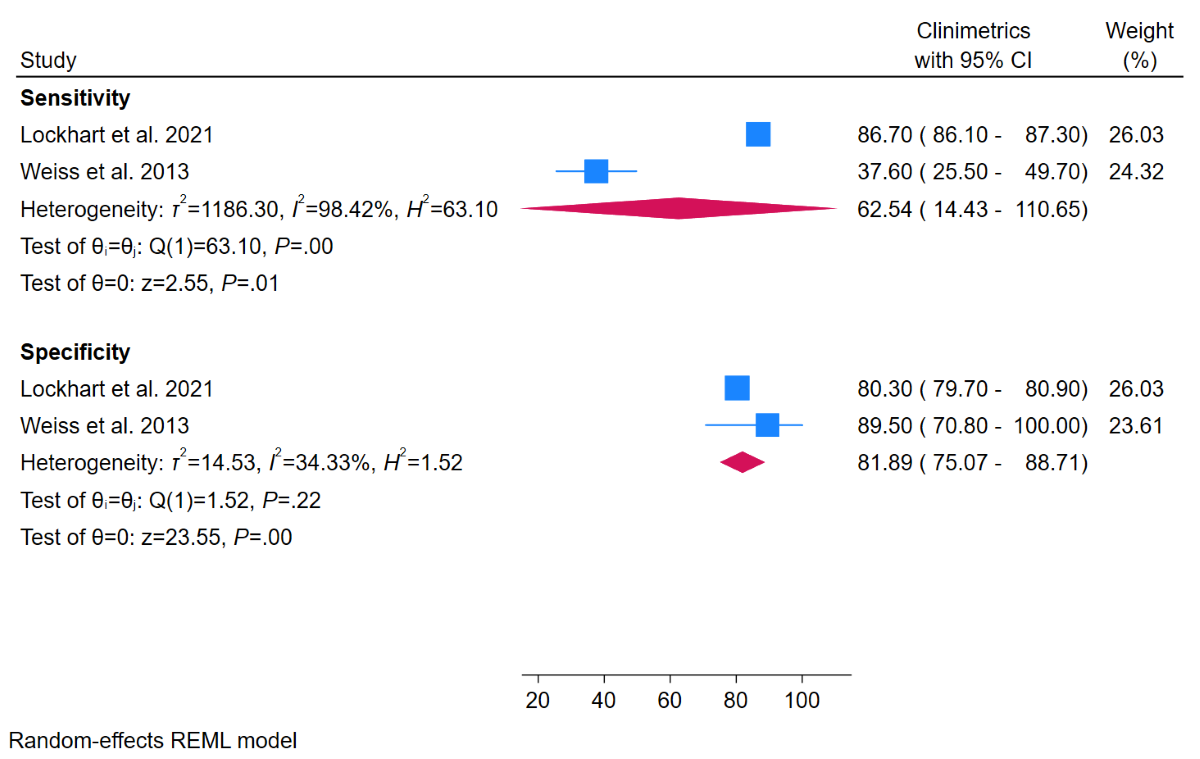
Meta-analyses of sensitivity and specificity of wearable activity trackers for detection for fall detection.

Meta-analysis results of sensitivity and specificity for fall detection are shown in Figure 5. Meta-analyses of 2 studies showed a specificity of 62.54% (95% CI 14.43%-100%) and a sensitivity of 81.89% (95% CI 75.07%-88.17%). There was an insufficient number of studies for subgroup analyses of device type and sensitivity analyses for fall detection.

Grade of recommendation: (D) inconsistent or inconclusive studies of any level for the use of wearable activity trackers to predict falls.

## Discussion

### Principal Findings

In this study, we set out to systematically review and meta-analyze the current evidence regarding wearable activity trackers’ ability to detect medical conditions and events under free-living conditions. To date, the majority of studies have focused on the detection of COVID-19, with a smaller number of studies focused on cardiac conditions and falls. For COVID-19 detection, the devices generally demonstrated good sensitivity and specificity. The most promising results were found for the detection of atrial fibrillation, for which the wearables showed high sensitivity and specificity. Whereas, for fall detection, the present findings devices showed moderate sensitivity but lower specificity. These findings indicate that while these devices are becoming more dependable for monitoring specific health conditions, their performance varies depending on the condition being detected.

The current body of evidence on the diagnostic potential of wearable activity trackers is notably skewed toward COVID-19 detection, a focus that is understandable given the pandemic’s global impact and the consequent urgent need for monitoring solutions. Researching the feasibility of detecting COVID-19 through wearables holds appeal due to the availability of widely used reference standards. Rapid and polymerase chain reaction tests, widely used, allow for easy self-reporting of COVID-19 diagnoses by many individuals. In contrast, accessing a reliable gold standard for other health outcomes poses significant challenges. However, what was surprising to note is the limited number of studies exploring these trackers for other health conditions, especially given that numerous wearables advertise features such as sleep apnea detection—a topic noticeably absent in our findings. Our extensive database search identified only a handful of studies each related to cardiac issues and falls. This gap in the literature is striking considering the wide array of health conditions that could theoretically be monitored using wearable technology, given their ability to capture data related to heart rate, movement, skin temperature, and more. Such capabilities would suggest that a broad spectrum of medical conditions could be measured, spanning cardiovascular and respiratory conditions to neurological and psychological disorders. It is important to note that we intentionally focused on the accuracy of data collected in free-living conditions (with a view to understanding current-day diagnostic capabilities). We note numerous laboratory-based studies that were excluded (eg, [24] and [25]) suggesting that a wider range of diagnostic outcomes may become available in the future. Furthermore, many studies were excluded because they focused on monitoring symptoms in people with a known diagnosis (eg, seizures in people with epilepsy [26], and freezing gait in Parkinson disease [27]) which was outside the scope of this study, but highlights wearable activity trackers’ potential for medical condition monitoring.

This study revealed that wearable activity trackers demonstrate moderate to high sensitivity and specificity for COVID-19 detection. It is interesting to compare our results with those for other COVID-19 screening tests. A systematic review by Mistry et al [28] on lateral flow devices (LFD) tests (also known as rapid antigen tests) evaluated 24 papers across 8 different LFD brands, covering over 26,000 test results. Their findings indicated that sensitivity ranged from 37.7% to 99.2% and specificity ranged from 92.4% to 100% [28]. Comparatively, this study’s pooled sensitivity for wearable-detected COVID-19 was 79.5% (range 51.3%-100%), which is in line with the LFD results. However, our specificity of 76.8% (range 63%-90.6%) was slightly lower. According to UK government guidelines, the benchmarks for COVID-19 workplace screening are ≥68% for sensitivity and ≥97% for specificity [29]. This suggests that while wearable activity monitor detection meets the sensitivity criterion, it falls short on specificity.

The most promising results were observed for the detection of atrial fibrillation, with figures that compare favorably to other clinical tests. For example, the sensitivity and specificity of a 12-lead electrocardiogram for detecting atrial fibrillation have previously been shown to range between 93% and 97% [30,31], which appears similar to our sensitivity and specificity of 94.2% and 95.3%, respectively. Over the course of 2022-2023, major brands, such as Fitbit [32], Apple Watch [33], Garmin [34], and Samsung [35], received approval from the US Food and Drug Administration for their atrial fibrillation detection features. The relatively higher accuracy in identifying cardiac arrhythmias as compared to COVID-19 is perhaps expected, given that cardiac functions can be deduced from wearables’ optical heart-rate sensors. In contrast, COVID-19 detection usually requires intricate algorithms that amalgamate multiple data points [36,37].

While wearable activity trackers demonstrated effectiveness in detecting cardiac arrhythmia and COVID-19, our meta-analysis revealed that their accuracy in detecting falls was only moderate. The devices were generally effective in identifying actual falls, with a sensitivity of 81.9%. However, they also generated a significant number of false positives, as evidenced by a lower specificity of 62.5%. This aligns with existing literature on the subject [38,39]. It is crucial to note that our review specifically focused on the performance of these devices in real-world conditions among the general population. Most existing studies on fall detection with wearables have been conducted in controlled laboratory settings using simulated falls, where accuracy has generally been higher [38,39]. The false positives in fall detection are likely due to the devices relying on accelerometry data, which can misinterpret other rapid downward movements as falls. Further research is needed to refine the algorithms used in these devices to improve their performance in fall detection. Future studies might incorporate additional metrics, such as rapid changes in heart rate or galvanic skin response, which may accompany a fall, to enhance accuracy.

This study offers several significant strengths, including being the first systematic review and meta-analysis focused on the real-world accuracy of wearable activity trackers in detecting medical conditions and events. The review analyzed a robust data set from 28 studies, involving over 1 million participants, enabling a comprehensive meta-analysis of various outcomes. Instead of limiting our focus to specific diagnostic outcomes, we examined a broad range of medical conditions. Our search strategy was exceptionally thorough, encompassing 10 databases and reviewing over 21,000 studies to capture a wide array of diagnostic outcomes. Methodologically, we adhered to the PRISMA 2020 guidelines, which included conducting sensitivity and subgroup analyses, as well as evaluating the certainty of the evidence.

Study limitations must be acknowledged. There was considerable heterogeneity in the designs of included studies, such as their reference standards, diagnostic tests, and sample characteristics. Given the size of the current evidence, there were too few studies to conduct separate subgroup analyses based on specific device models or software versions. Our review included both researcher-initiated and consumer-initiated studies to provide a comprehensive assessment of wearable activity trackers in real-world settings. Researcher-initiated studies typically involved smaller sample sizes and controlled participant recruitment, while consumer-initiated studies often had larger sample sizes and reflected more naturalistic use patterns. While this combination enhances the generalizability of our findings, it also introduces heterogeneity. We acknowledge this as a limitation and suggest that future research should consider these differences when interpreting results. Additionally, our review only identified studies in the domains of COVID-19, cardiovascular conditions, and falls as eligible. While laboratory-based studies are being conducted for event detection in other health domains (such as stress and respiratory conditions), our focus was intentionally on studies conducted in free-living conditions. This approach offers insights into the wearables’ event detection capabilities in real-world settings, as opposed to artificial (eg, laboratory) conditions.

### Clinical Implications

The use of wearable activity trackers for detecting medical events is an emerging field with both significant promise and challenges. Wearable activity trackers demonstrate comparable ability to detect COVID-19 and atrial fibrillation compared with other clinical tests such as lateral flow tests and electrocardiograms. However, wearables offer the additional advantage of continuous, real-time monitoring for conditions requiring constant surveillance. As such, they may empower patients to take a more proactive role in their health care by giving them immediate feedback and data about their condition. They may also contribute to improved surveillance and resource planning for health care systems, which could be particularly useful in times of epidemics or pandemics.

Certain wearable device features excel at detecting specific medical events. For COVID-19, devices combining heart rate monitors, skin temperature sensors, and accelerometers proved effective by detecting deviations from an individual’s baseline across multiple physiological parameters. In contrast, for atrial fibrillation detection, Food and Drug Administration-approved devices relied on optical heart rate sensors providing photoplethysmography data, capable of identifying irregular heart rhythms characteristic of arrhythmias. Fall detection primarily uses accelerometer data, with wrist-worn placement crucial for sensing sudden deceleration and impact forces. However, false positives persist due to nonfall rapid movements. Looking ahead, integrating multiple sensors can enhance accuracy across various medical conditions. Yet, fundamental sensor limitations may remain. Aligning device capabilities with specific use cases and recognizing sensor shortcomings will inform future research and benchmarking efforts amid evolving technology.

As consumer wearables gradually morph from being lifestyle tools to over-the-counter medical instruments, they present a range of challenges, including concerns about data privacy and security, which will require stringent protective measures. Furthermore, as wearable devices become increasingly sophisticated in detecting medical conditions, such as atrial fibrillation, they offer both benefits and pitfalls. On the positive side, these devices have the potential to identify asymptomatic atrial fibrillation episodes. This is enormously beneficial, since currently, stroke is the first manifestation in at least 25% of atrial fibrillation-related stroke cases [40]. Early detection could therefore lead to timely intervention and stroke prevention. However, health care professionals have reported an uptick in patient consultations triggered by atrial fibrillation alerts from wearables, resulting in a surge of medical tests, such as electrocardiograms, to confirm diagnoses [41]. While some clinicians see this as an advancement in patient-initiated health care, others question the necessity of such screening, particularly in patient subgroups where atrial fibrillation may have a relatively benign prognosis [42]. Moreover, the use of wearables can generate both false positives and negatives, potentially causing unnecessary anxiety, diagnostic tests, and treatments, or giving users a false sense of security.

### Future Research

Our review reveals that the current peer-reviewed evidence base concerning the event detection capabilities of consumer wearable activity trackers in free-living conditions is limited to COVID-19, cardiac function, and falls. This was somewhat surprising, given the potential of these devices to diagnose numerous other conditions. Our findings indicate a significant gap in the current literature, which was not apparent in previous reviews that typically focused on specific conditions and did not highlight the lack of studies across a broader range of conditions. Considering the diverse array of sensors incorporated in modern wearable activity trackers, these devices offer considerable potential for detecting and monitoring medical events across an extensive spectrum of health conditions into the future. This may include respiratory conditions, neurological disorders, mental health, stress and fatigue, and even environmental and allergic reactions. This will require research across the product design continuum, from algorithm training to laboratory testing and free-living testing. This will be made all the more challenging by the rapid pace at which new devices and models are released into the market. In the future, our meta-analysis could be updated to provide insight into the accuracy of such diagnostics by condition, device, and population.

### Conclusions

This study provides a comprehensive overview of the current state of evidence regarding the diagnostic capabilities of consumer wearable activity trackers in real-world settings. While the devices show promise in detecting conditions, such as COVID-19 and atrial fibrillation, with moderate to high sensitivity and specificity, their performance in detecting falls is moderate, highlighting the need for further refinement of detection algorithms. The existing literature is notably skewed toward COVID-19, leaving a significant gap in our understanding of how these devices can be used for a broader range of health issues. This gap, which was not apparent in previous reviews, underscores the necessity for future research to expand the scope of conditions studied. As wearable technology continues to evolve, it is crucial to address the challenges posed by false positives and negatives, data privacy, and security concerns. This will ensure that the rapid advancements in this field can be matched by robust scientific validation, enabling these devices to realize their full potential as tools for health care monitoring and intervention.

## Appendix

Multimedia Appendix 1 Supplementary materials.

Multimedia Appendix 2 Preferred Reporting Items for Systematic Review and Meta-Analysis (PRISMA) checklist.

## Abbreviations

AUC: area under the curve
JBI: Joanna Briggs Institute
LFD: lateral flow device
MeSH: Medical Subject Headings
PPV: positive predictive value
PRISMA: Preferred Reporting Items for Systematic Reviews and Meta-Analyses

## Data Availability

All data generated or analyzed during this study are included in this published article and Multimedia Appendix 1.
