# Supplementary material for: Real-World Accuracy of Wearable Activity Trackers for Detecting Medical Conditions: Systematic Review and Meta-Analysis
Source: JMIR Mhealth Uhealth. 2024 Aug 30;12:e56972. doi: 10.2196/56972 (PMC11399740; doi:10.2196/56972)
Supplement: Multimedia Appendix 1 [file mhealth_v12i1e56972_app1.docx]

# **Multimedia Appendix 1.** Supplementary material.

# Table S1. Overview of study inclusion criteria

| **Criteria** | **Inclusion** | **Exclusion** |
| --- | --- | --- |
| Population | - Adult population (aged 18 years or older) in free-living conditions, not recruited based on specific health condition or diagnosis. | - Studies involving children or adolescents. |
| Exposure | - Use of a wearable activity tracker (e.g., Fitbit, Apple Watch, or research-grade accelerometer). - Wearable activity tracker able to detect movement behavior (e.g., includes an accelerometer), may include other sensors. - Wearable activity tracker consists of a single device worn on a single body location (e.g., wrist or chest). | - Wearable devices unable to track activity levels (e.g., continuous glucose monitors). - Studies examining symptoms within people known to have a specific disease. - Studies evaluating an array of wearable sensors worn at multiple body locations (e.g., watch plus skin patch) or pedometers. |
| Outcomes | - Studies assessing actual diagnosis of medical condition or occurrence of clinically relevant events (e.g., falls). - Studies reporting outcomes related to diagnostic accuracy, such as specificity and/or sensitivity of the device for early detection of disease or medical events. | - Studies measuring association between exposure and outcome using odds ratios, relative risk, hazard ratios. - Lab- or hospital-based studies. - Conference abstracts or dissertations. |
| Study Type | - Validation studies conducted under free-living conditions reported in peer-reviewed journal articles. - Secondary analyses within observational, experimental, or quasi-experimental studies. |  |

# Table S2. Medline search strategy and terms. Note: these search terms were adapted for the other databases

| MEDLINE(R) ALL <1946 to June 19, 2023>  Ovid MEDLINE(R) ALL <1946 to June 19, 2023> | |
| --- | --- |
| 1. accelerometry 2. acceleromet* 3. actigraphy 4. actigraph 5. wearable electronic devices 6. fitness trackers 7. activity monitor* 8. activity tracker* 9. acceleromet* 10. wearable* 11. fitness tracker* 12. fitness monitor* 13. Fitbit 14. Apple watch 15. Garmin 16. Samsung 17. Galaxy 18. iFit 19. Google Pixel watch 20. Halo view 21. Polar 22. Nike fuelband 23. Withings 24. Whoop 25. MiCoach | 1. Oura Ring 2. NFC OPN Smart Ring 3. Ticwatch 4. Huawei Band 5. Amazfit 6. Jawbone 7. Honor 8. Coros Apex 9. sensewear* 10. xiaomi* 11. Go2Sleep 12. ((fit* or activity) adj1 (monitor* OR track* OR sens* OR band*)).mp. 13. ((smart or sport*) adj1 watch)).mp. 14. early diagnosis[MeSH] 15. detect* 16. diagnos* 17. predict* 18. identif* 19. screening 20. future onset 21. follow-up |

# Table S3. List of full-text exclusions with reasons.

|  | Title | First author | Year | Journal | Reason |
| --- | --- | --- | --- | --- | --- |
| 1 | Unobtrusive Sensing and Wearable Devices for Health Informatics | Zheng | 2014 | IEEE Transactions on Biomedical Engineering | Wrong study design |
| 2 | Remote health monitoring of elderly through wearable sensors | Al-khafajiy | 2019 | Multimedia Tools and Applications | Wrong study design |
| 3 | Unsynchronized wearable sensor data analytics model for improving the performance of smart healthcare systems | Alfarraj | 2021 | Journal of Ambient Intelligence and Humanized Computing | Wrong study design |
| 4 | An intelligent healthcare monitoring framework using wearable sensors and social networking data | Ali | 2021 | Future Generation Computer Systems | Wrong study design |
| 5 | Covariance matrix-based fall detection from multiple wearable sensors | Boutellaa | 2019 | Journal of Biomedical Informatics | Wrong study design |
| 6 | Remote Design of a Smartphone and Wearable Detected Atrial Arrhythmia in Older Adults Case Finding Study: Smart in OAC - AFNET 9 | Fabritz | 2022 | Frontiers in Cardiovascular Medicine | Wrong study design |
| 7 | Predicting falls in people with dementia using accelerometry:A one-year prospective multi-center field study | Gietzelt | 2014 | Biomedizinische Technik/Biomedical Engineering | Wrong study design |
| 8 | The Design and Implementation of Mobile Heart Monitoring Applications using Wearable Heart Rate Sensor | Hashim | 2021 | International Journal of Advanced Computer Science and Applications | Wrong study design |
| 9 | Detection of fall for the elderly in an indoor environment using a tri-axial accelerometer and Kinect depth data | Jansi | 2020 | Multidimensional Systems and Signal Processing | Wrong study design |
| 10 | Wearable non-invasive monitors of diabetes and hypoxia through continuous analysis of sweat | Karpova | 2020 | Talanta | Wrong study design |
| 11 | Contextual activity-based Healthcare Internet of Things, Services, and People (HIoTSP): An architectural framework for healthcare monitoring using wearable sensors | Khowaja | 2018 | Computer Networks | Wrong study design |
| 12 | Automated Detection of Convulsive Seizures Using a Wearable Accelerometer Device | Kusmakar | 2019 | IEEE Trans Biomed Eng | Wrong study design |
| 13 | Wearable Armband Device for Daily Life Electrocardiogram Monitoring | Lazaro | 2020 | IEEE Trans Biomed Eng | Wrong study design |
| 14 | Design and implementation of wearable medical monitoring system on the internet of things | Li | 2021 | Journal of Ambient Intelligence and Humanized Computing | Wrong study design |
| 15 | Machine learning detection of Atrial Fibrillation using wearable technology | Lown | 2020 | PLoS One | Wrong study design |
| 16 | Accelerometer-based quantitative analysis of axial nocturnal movements differentiates patients with Parkinson’s disease, but not high-risk individuals, from controls | Maartje | 2015 | Journal of Neurology, Neurosurgery Psychiatry | Wrong study design |
| 17 | Fall detection and activity identification using wearable and hand-held devices | Maglogiannis | 2016 | Integrated Computer-Aided Engineering | Wrong study design |
| 18 | Learning from Large-Scale Wearable Device Data for Predicting the Epidemic Trend of COVID-19 | Zhu | 2020 | Discrete Dynamics in Nature and Society | Wrong study design |
| 19 | Online Fall Detection Using Recurrent Neural Networks on Smart Wearable Devices | Musci | 2021 | IEEE Transactions on Emerging Topics in Computing | Wrong study design |
| 20 | IOT based wearable sensor for diseases prediction and symptom analysis in healthcare sector | Muthu | 2020 | Peer-to-Peer Networking and Applications | Wrong study design |
| 21 | Harnessing wearable device data to improve state-level real-time surveillance of influenza-like illness in the USA: a population-based study | Radin | 2020 | The Lancet Digital Health | Wrong study design |
| 22 | Smart wearable model for predicting heart disease using machine learning | Rani | 2022 | Journal of Ambient Intelligence and Humanized Computing | Wrong study design |
| 23 | Adapted step length estimators for patients with Parkinson's disease using a lateral belt worn accelerometer | Sayeed | 2015 | Technol Health Care | Wrong study design |
| 24 | An accelerometry-based study of lower and upper limb tremor in Parkinson's disease | Scanlon | 2013 | J Clin Neurosci | Wrong study design |
| 25 | A method for intelligent allocation of diagnostic testing by leveraging data from commercial wearable devices: a case study on COVID-19 | Shandhi | 2022 | npj Digital Medicine | Wrong study design |
| 26 | Real-Time Quality Assessment of Long-Term ECG Signals Recorded by Wearables in Free-Living Conditions | Smital | 2020 | IEEE Transactions on Biomedical Engineering | Wrong study design |
| 27 | Tracking generalized tonic-clonic seizures with a wrist accelerometer linked to an online database | Velez | 2016 | Seizure | Wrong study design |
| 28 | Elderly Fall Detection with an Accelerometer Using Lightweight Neural Networks | Wang | 2019 | Electronics | Wrong study design |
| 29 | CMFALL: A Cascade and Parallel Multi-State Fall Detection Algorithm Using Waist-Mounted Tri-Axial Accelerometer Signals | Wang | 2020 | IEEE Transactions on Consumer Electronics | Wrong study design |
| 30 | Development of a wearable-sensor-based fall detection system | Wu | 2015 | Int J Telemed Appl | Wrong study design |
| 31 | Fall Detection with Wearable Sensors: A Hierarchical Attention-based Convolutional Neural Network Approach | Yu | 2021 | Journal of Management Information Systems | Wrong study design |
| 32 | Unobtrusive Sensing and Wearable Devices for Health Informatics | Zheng | 2014 | IEEE Transactions on Biomedical Engineering | Wrong study design |
| 33 | Detecting freezing of gait with a tri-axial accelerometer in Parkinson's disease patients | Ahlrichs | 2016 | Med Biol Eng Comput | Wrong setting |
| 34 | Detection of generalized tonic-clonic seizures by a wireless wrist accelerometer: a prospective, multicenter study | Beniczky | 2013 | Epilepsia | Wrong setting |
| 35 | Foreseeing future falls with accelerometer features in active community-dwelling older persons with no recent history of falls | Bet | 2021 | Exp Gerontol | Wrong setting |
| 36 | Abnormal respiratory event detection in sleep: A prescreening system with smart wearables | Camca | 2019 | Journal of Biomedical Informatics | Wrong setting |
| 37 | Wearable IoT based diagnosis of prostate cancer using GLCM-multiclass SVM and SIFT-multiclass SVM feature extraction strategies | Chandrasekhara | 2021 | International Journal of Pervasive Computing and Communications | Wrong setting |
| 38 | Real-time infection prediction with wearable physiological monitoring and AI to aid military workforce readiness during COVID-19 | Conroy | 2022 | Scientific Reports | Wrong setting |
| 39 | A Scalable Risk-Scoring System Based on Consumer-Grade Wearables for Inpatients With COVID-19: Statistical Analysis and Model Development | Foll | 2022 | JMIR Form Res | Wrong setting |
| 40 | Matching pursuit-based compressive sensing in a wearable biomedical accelerometer fall diagnosis device | Gibson | 2017 | Biomedical Signal Processing and Control | Wrong setting |
| 41 | Precision wearable accelerometer contact microphones for longitudinal monitoring of mechano-acoustic cardiopulmonary signals | Gupta | 2020 | npj Digital Medicine | Wrong setting |
| 42 | Quantitative detection of sleep apnea with wearable watch device | Hayano | 2020 | PLoS One | Wrong setting |
| 43 | Associative Classification based Human Activity Recognition and Fall Detection using Accelerometer | Hemalatha | 2013 | International Journal of Intelligent Information Technologies | Wrong setting |
| 44 | Tonic-clonic seizure detection using accelerometry-based wearable sensors: A prospective, video-EEG controlled study | Johansson | 2019 | Seizure | Wrong setting |
| 45 | Improved screening of fall risk using free-living based accelerometer data | Kelly | 2022 | J Biomed Inform | Wrong setting |
| 46 | A novel fall detection algorithm for elderly using SHIMMER wearable sensors | Mehmood | 2019 | Health and Technology | Wrong setting |
| 47 | Machine learning from wristband sensor data for wearable, noninvasive seizure forecasting | Meisel | 2020 | Epilepsia | Wrong setting |
| 48 | Autonomous identification of freezing of gait in Parkinson's disease from lower-body segmental accelerometry | Moore | 2013 | Journal of NeuroEngineering and Rehabilitation | Wrong setting |
| 49 | Development of a Knowledge Discovery Computing based wearable ECG monitoring system | Noh | 2020 | Information Technology and Management | Wrong setting |
| 50 | Multicenter clinical assessment of improved wearable multimodal convulsive seizure detectors | Onorati | 2017 | Epilepsia | Wrong setting |
| 51 | Detection of seizure-like movements using a wrist accelerometer | Lockman | 2011 | Epilepsy Behav | Wrong setting |
| 52 | Pre-Impact Fall Detection Based on Wearable Device Using Dynamic Threshold Model | Otanasap | 2016 | 2016 17th International Conference on Parallel and Distributed Computing, Applications and Technologies (PDCAT) | Wrong setting |
| 53 | Detecting sleep using heart rate and motion data from multisensor consumer-grade wearables, relative to wrist actigraphy and polysomnography | Roberts | 2020 | Sleep | Wrong setting |
| 54 | A Patient-Specific Single Sensor IoT-Based Wearable Fall Prediction and Detection System | Saadeh | 2019 | IEEE Transactions on Neural Systems and Rehabilitation Engineering | Wrong setting |
| 55 | The Predictive Performance of Objective Measures of Physical Activity Derived From Accelerometry Data for 5-Year All-Cause Mortality in Older Adults: National Health and Nutritional Examination Survey 2003-2006 | Smirnova | 2020 | J Gerontol A Biol Sci Med Sci | Wrong setting |
| 56 | Using PPG Signals and Wearable Devices for Atrial Fibrillation Screening | Yang | 2019 | IEEE Transactions on Industrial Electronics | Wrong setting |
| 57 | An Energy-Efficient Algorithm for Classification of Fall Types Using a Wearable Sensor | Kwon | 2019 | IEEE Access | Wrong setting |
| 58 | Complexity index from a personalized wearable monitoring system for assessing remission in mental health | Lanata | 2015 | IEEE J Biomed Health Inform | Wrong setting |
| 59 | Continuous Wearable Monitoring Analytics Predict Heart Failure Hospitalization: The LINK-HF Multicenter Study | Stehlik | 2020 | Circ Heart Fail | Wrong setting |
| 60 | Personalized approach using wearable technology for early detection of atrial fibrillation in high-risk primary care patients (PATCH-AF): Study protocol for a cluster randomized controlled trial | Brik | 2022 | American Heart Journal | Ineligible wearable |
| 61 | Using Consumer-Wearable Activity Trackers for Risk Prediction of Life-Threatening Heart Arrhythmia in Patients with an Implantable Cardioverter-Defibrillator: An Exploratory Observational Study | Frodi | 2022 | J Pers Med | Ineligible wearable |
| 62 | Feasibility of a T-Shirt-Type Wearable Electrocardiography Monitor for Detection of Covert Atrial Fibrillation in Young Healthy Adults | Fukuma | 2019 | Scientific Reports | Ineligible wearable |
| 63 | Machine learning detection of obstructive hypertrophic cardiomyopathy using a wearable biosensor | Green | 2019 | npj Digital Medicine | Ineligible wearable |
| 64 | Automatic Detection and Classification of Convulsive Psychogenic Nonepileptic Seizures Using a Wearable Device | Gubbi | 2016 | IEEE J Biomed Health Inform | Ineligible wearable |
| 65 | Screening of obstructive sleep apnea in patients who snore using a patch-type device with electrocardiogram and 3-axis accelerometer | Hsu | 2020 | J Clin Sleep Med | Ineligible wearable |
| 66 | Clinical Implications of Atrial Fibrillation Detection Using Wearable Devices in Patients With Cryptogenic Stroke (CANDLE-AF) Trial: Design and Rationale | Jung | 2022 | Front Cardiovasc Med | Ineligible wearable |
| 67 | Event-driven system for fall detection using body-worn accelerometer and depth sensor | Kepski | 2018 | IET Computer Vision | Ineligible wearable |
| 68 | Deep Learning Based Fall Detection Algorithms for Embedded Systems, Smartwatches, and IoT Devices Using Accelerometers | Kraft | 2020 | Technologies | Ineligible wearable |
| 69 | Wearable skin-like optoelectronic systems with suppression of motion artifacts for cuff-less continuous blood pressure monitor | Li | 2020 | National Science Review | Ineligible wearable |
| 70 | Automated Classification of Atrial Fibrillation Using Artificial Neural Network for Wearable Devices | Ma | 2020 | Mathematical Problems in Engineering | Ineligible wearable |
| 71 | An Energy Efficient Wearable Smart IoT System to Predict Cardiac Arrest | Majumder | 2019 | Advances in Human-Computer Interaction | Ineligible wearable |
| 72 | A novel approach for IoT based wearable health monitoring and messaging system | Manas | 2019 | Journal of Ambient Intelligence and Humanized Computing | Ineligible wearable |
| 73 | Chest Wearable Apparatus for Cuffless Continuous Blood Pressure Measurements Based on PPG and PCG Signals | Marzorati | 2020 | IEEE Access | Ineligible wearable |
| 74 | A wearable device for physical and emotional health monitoring | Murali | 2015 | Computing in Cardiology | Ineligible wearable |
| 75 | Towards an Accelerometer-Based Elderly Fall Detection System Using Cross-Disciplinary Time Series Features | Nahian | 2021 | IEEE Access | Ineligible wearable |
| 76 | Accelerometric patch probe for cuffless blood pressure evaluation from carotid local pulse wave velocity: design, development, and in-vivo experimental study | Rayirath | 2019 | Biomedical Physics & Engineering Express | Ineligible wearable |
| 77 | Acoustic Sensing as a Novel Wearable Approach for Cardiac Monitoring at the Wrist | Sharma | 2019 | Scientific Reports | Ineligible wearable |
| 78 | A wearable real-time telemonitoring electrocardiogram device compared with traditional Holter monitoring | Shen | 2020 | J Biomed Res | Ineligible wearable |
| 79 | Arm-ECG Wireless Sensor System for Wearable Long-Term Surveillance of Heart Arrhythmias | Villegas | 2019 | Electronics | Ineligible wearable |
| 80 | Comparative study of a wearable intelligent sleep monitor and polysomnography monitor for the diagnosis of obstructive sleep apnea | Xu | 2023 | Sleep Breath | Ineligible wearable |
| 81 | A Wearable Fall Detection System based on LoRa LPWAN Technology | Zanaj | 2020 | Journal of Communications Software and Systems | Ineligible wearable |
| 82 | Novel Use of Apple Watch 4 to Obtain 3-Lead Electrocardiogram and Detect Cardiac Ischemia | Avila | 2019 | Perm J | Wrong outcomes |
| 83 | Watch-Dog: Detecting Self-Harming Activities From Wrist Worn Accelerometers | Bharti | 2018 | IEEE J Biomed Health Inform | Wrong outcomes |
| 84 | Patients self-mastery of wearable devices for seizure detection: A direct user-experience | Bruno | 2020 | Seizure | Wrong outcomes |
| 85 | Deep learning with wearable based heart rate variability for prediction of mental and general health | Coutts | 2020 | Journal of Biomedical Informatics | Wrong outcomes |
| 86 | Use of Physiological Data From a Wearable Device to Identify SARS-CoV-2 Infection and Symptoms and Predict COVID-19 Diagnosis: Observational Study | Hirten | 2021 | J Med Internet Res | Wrong outcomes |
| 87 | Utilizing daily mood diaries and wearable sensor data to predict depression and suicidal ideation among medical interns | Horwitz | 2022 | J Affect Disord | Wrong outcomes |
| 88 | Predicting Subjective Recovery from Lower Limb Surgery Using Consumer Wearables | Karas | 2020 | Digital Biomarkers | Wrong outcomes |
| 89 | Fall detection and human activity classification using wearable sensors and compressed sensing | Kerdjidj | 2020 | Journal of Ambient Intelligence and Humanized Computing | Wrong outcomes |
| 90 | Rapid Screening of Physiological Changes Associated With COVID-19 Using Soft-Wearables and Structured Activities: A Pilot Study | Lonini | 2021 | IEEE Journal of Translational Engineering in Health and Medicine | Wrong outcomes |
| 91 | Deep longitudinal phenotyping of wearable sensor data reveals independent markers of longevity, stress, and resilience | Pyrkov | 2021 | Aging (Albany NY) | Wrong outcomes |
| 92 | Wearable devices can predict the outcome of standardized 6-minute walk tests in heart disease | Schubert | 2020 | npj Digital Medicine | Wrong outcomes |
| 93 | Arrhythmias Other Than Atrial Fibrillation in Those With an Irregular Pulse Detected With a Smartwatch: Findings From the Apple Heart Study | Perino | 2021 | Circulation: Arrhythmia and Electrophysiology logo | Wrong outcomes |
| 94 | Prediction model development of women's daily asthma control using fitness tracker sleep disruption | Castner | 2020 | Heart Lung | Wrong population |
| 95 | A Bipolar Disorder Monitoring System Based on Wearable Device and Smartphone | Coelho | 2016 | IFAC-PapersOnLine | Wrong population |
| 96 | Diagnosis of Atrial Fibrillation Using Machine Learning With Wearable Devices After Cardiac Surgery: Algorithm Development Study | Hiraoka | 2022 | JMIR Form Res | Wrong population |
| 97 | Seizure detection based on heart rate variability using a wearable electrocardiography device | Jeppesen | 2019 | Epilepsia | Wrong population |
| 98 | Feasibility Study of Monitoring Deterioration of Outpatients Using Multimodal Data Collected by Wearables | Li | 2020 | ACM Trans. Comput. Healthcare | Wrong population |
| 99 | In-hospital and home-based long-term monitoring of focal epilepsy with a wearable electroencephalographic device: Diagnostic yield and user experience | Macea | 2023 | Epilepsia | Wrong population |
| 100 | The usefulness of accelerometric registration with assessment of tremor parameters and their symmetry in differential diagnosis of parkinsonian, essential and cerebellar tremor | Machowska-Majchrzak | 2012 | Neurologia i Neurochirurgia Polska | Wrong population |
| 101 | Evaluation of an Activity Tracker to Detect Seizures Using Machine Learning | Mittlesteadt | 2020 | J Child Neurol | Wrong population |
| 102 | Convulsive seizure detection using a wrist-worn electrodermal activity and accelerometry biosensor | Poh | 2012 | Epilepsia | Wrong population |
| 103 | Automated assessment of pulmonary patients using heart rate variability from everyday wearables | Rahman | 2020 | Smart Health | Wrong population |
| 104 | Potential roles of the wearable cardioverter-defibrillator in acute phase care of patients at high risk of sudden cardiac death: A single-center Japanese experience | Sasaki | 2017 | J Cardiol | Wrong population |
| 105 | Evaluating depression with multimodal wristband-type wearable device: screening and assessing patient severity utilizing machine-learning | Tazawa | 2020 | Heliyon | Wrong population |

# Table S4. Overview of characteristics of included studies (n=28).

| Author, year | Country | Sample size, gender | Age | Wearable device | Data Input/ Vital Signs Collected | Disease or medical event | Data Labelling/Data Segmentation | Reference standard | Algorithm or statistical model | Algorithm/accelerometer data open or available to access? |
| --- | --- | --- | --- | --- | --- | --- | --- | --- | --- | --- |
| Abir 2022 | Qatar | 120 Gender: NR | NR | Fitbit | Heart rate, steps | COVID-19 | At least 20 days before the symptom onset to 21 days afterward. | Reverse transcription-polymerase chain reaction | Long Short-term Memory Variational Autoencoder-based anomaly sdetection framework | No /Yes |
| Alavi 2022 | United States | 3,318 F: 1,854 (56%) M: 1,464 (44%) | Median (range): 44 (19-79) | Fitbit, Apple Watch & Garmin | Heart rate, steps | COVID-19 | 3 days before symptom onset, for the next 15 days. | COVID-19 diagnosis for asymptomatic cases and self reported symptom for symptomatic individuals | NightSignal, RHRAD, CuSum | Yes /Yes |
| Caillol 2021 | France | 256 F: 108 (42%) M: 148 (58%) | 66±6 | Apple Watch | Sinus rhythm | Arrhythmia | NR | 12-lead ECG | Cohen’s kappa | No /No |
| Cleary 2022 | United States | 105 F: 53 (51%) M: 52 (49%) | 28.6±2.8 | Fitbit & Apple watch | Heart rate, steps and sleep | COVID-19 | 0-7 days after symptom onset as test periods. | COVID-19-positive test | RHRmetric, SLEEPmetric, STEPmetric, SENSORmetric | Yes /Yes |
| D’Haese 2021 | United States | 867 F: 313 (36.1%) M: 236 (27.2%) Unknown: 318 (36.7%) | 37.6±11.3 | Oura Ring | Body temperature, sleep, activity, heart rate, respiratory rate, heart rate variability | Viral symptoms | 3 days before symptom onset | Self-reported viral illness | Labelling model and forecasting model | Yes /No |
| Gadaleta 2021 | United States | 38,911 F: 23,736 (61%) M: 15,175 (39%) | NR | Fitbit & Apple Watch | Heart rate, sleep, activity | COVID-19 | NR | COVID-19 nasal swab test | CatBoost gradient boosting | Yes /Yes |
| Guo 2019 | China | 187,912 F: 24,938 (13.3%) M: 16,2974 (86.7%) | 34.7±11.5 | Honor Band 4, Huawei Watch & Honor Watch | Pulse rhythm | Atrial Fibrillation | NR | Clinical evaluation, ECG, or 24-h Holter monitoring | Kruskal-Wallis test and a photoplethysmography algorithm | Yes /No |
| Hassantabar 2012 | Italy | 87 Gender: NR | NR | Empatica E4 | Galvanic skin response, temperature, interbeat interval, oxygen saturation | COVID-19 | NR | COVID-19-positive test | Naïve Bayes, Random Forest, Ada Boost, Decision Tree, SVM, k-NN, deep neural network model with grow-and-prune synthesis | Yes /No |
| Hirten 2021 | United States | 297 F: 204 (69.4%) M: 93 (31.3%) | 36.3±9.8 | Apple watch | Heart rate variability | COVID-19 | Defined being symptomatic as the first day of a reported symptom | COVID-19-positive nasal swab PCR test | Mixed-effect Cosinor model. | No /No |
| Liu 2021 | Europe | 87 F: 24 (27.6%) M: 63 (72.4%) | 46.5±10.5 | Fitbit | Heart rate | COVID-19 | NR | COVID-19 diagnosis | Conventional neural network, Multilayer Perceptrons, Long Short-Term Memory Networks, conventional convolutional auto-encoder, contrastive conventional convolutional auto-encoder | No /No |
| Lockhart 2021 | United States | 171 Gender: NR | Fallers: 75.4±8.7 Non-fallers: 75.7±7.6 | Inertial measurement unit (accelerometer worn on sternum) | Trunk kinematics | Falls | NR | Ten-meter walk test and fall history | Modified continuous wavelet transform method | Yes /No |
| Lonini 2020 | United States | 29 F: 11 (50%) M: 11 (50%) | 42.9±15.9 | Soft wearable sensor (accelerometer worn on suprasternal notch) | RR intervals, steps, RR and frequency spectrum of cough signals | COVID-19 | Labelled snapshots as COVID-19 positive and negative | COVID-19 diagnosis PCR test | Logistic Regression (Supervised machine learning) | No /No |
| Lubitz 2022 | United States | 455,699 F: 323,365 (71%) M: 132,334 (29%) | Median (IQR): 47 (35-58) | Fitbit | Heart rate | Atrial Fibrillation | NR | Single-lead ECG patch monitor | Fitbit PPG RhythmDetect Software System algorithm | No /No |
| Mason 2022 | United States | 63,153 F: 24,374 (40%) M: 36,632 (60%) Other: 56 (<0.1%) | 18-30 y: n=8,555 (14%) 31-40 y: n=16,756 (27%) 41-50y: n=17,502 (29%) 51-80y: n=18,148 (30%) 81+ y: n=102 (0.2%) | Oura Ring | Heart rate, heart rate variability, dermal temperature, respiratory rate, physical activity (METs) | COVID‑19 | NR | COVID-19 PCR test | Machine learning, random forest models | No /No |
| Mishra 2020 | United States | 5,262 32 diagnosed F: 25 (78.1%) M: 7 (21.9%) | Mean (range): 47 (27-67) | Fitbit | Heart rate, steps | COVID-19 | Dates of symptom onset and diagnosis to define sick periods | COVID-19 diagnosis | RHR-Diff, HROS-AD, CuSum | Yes /Yes |
| Natarajan 2020 | United States | 2,745 Gender: NR | 40.7±12.3 | Fitbit | RR, heart rate, and HRV | COVID-19 | Data from 2nd to 6^th^ day of symptom onset labelled as sick | COVID-19 diagnosis PCR test | CNN (Supervised deep learning) | No /No |
| Nestor 2023 | Canada | 6,926 F: 6,012 (86.8%) M: 914 (13.2%) | NR | Fitbit | Night-time RR, heart rate, HRV and symptom report | COVID-19 | Days between self reported symptom onset and self-reported recovery labelled as positive | COVID-19 diagnosis | XGBoost, XGBoost and GRU-D | No /Yes |
| Perez 2019 | United States | 419,297 F: 177,087 (42%) M: 238,700 (57%) Other: 396 (0.1%) NR: 3,114 (0.7%) | 41±13 | Apple Watch | Pulse rate | Atrial fibrillation | NR | ECG patch | Irregular Pulse Notification Algorithm | No /No |
| Ploux 2021 | France | 260 F: 109 (42%) M: 151 (58%) | 66±6 | Apple Watch | Sinus rhythm | Arrhythmia | NR | 12-lead ECG | NR | No /No |
| Quer 2021 | United States | 30,529 F: 18,922 (62%) M: 11, 607 (38%) | NR | Fitbit & Apple Watch | Heart rate, sleep, activity | COVID-19 | First date of symptoms to seven days after symptoms considered infectious | COVID-19 diagnosis | RHRMetric, SleepMetric, ActivityMetric, SymptomMetric, SensorMetric, OverallMetric | Yes /Yes |
| Skibińska 2021 | Czech Republic | 54 Gender: NR | NR | Fitbit, Apple Watch & various others | Heart rate, steps, sleep | COVID-19 | NR | COVID-19 diagnosis | XGBoost, k-NN, SVM, Logistic Regression Decision Tree Random Forest | Yes /Yes |
| Skibińska 2022 | Czech Republic | 58 Gender: NR | NR | Fitbit, Apple Watch & various others | Heart rate, steps, sleep | COVID-19 | NR | COVID-19 diagnosis | XGBoost, k-NN, Logistic Regression, Support Vector Machine (SVM), Decision Tree, and Random Forest. | Yes /Yes |
| Smarr 2020 | United States | 50 F: 22 (44%) M: 28 (66%) | 43.7±11.0 | Oura ring | Temperature, heart rate, heart rate variability, and respiration rate | Fever and COVID-19 | Onset of reporting of symptoms | COVID-19 diagnosis | Wavelet analysis | Yes /Yes |
| Tison 2018 | United States | 9,750 M: 5395 (55.3%) F: 2195 (22.5%) Unknown: 973 (10%) NR: 1187 (12.2%) | Atrial Fibrillation: 55.7±14.2 No Atrial Fibrillation: 41.5 ±11.9 | Apple watch | Heart rate, step count, R-R interval | Atrial Fibrillation | NR | 12-lead ECG | Deep neural network development and training | Yes /No |
| van Schooten 2016 | Netherlands | 319 F: 161 (50.5%) M: 158 (49.5%) | 75.5±6.9 | Dynaport MoveMonitor accelerometer | Daily-life gait quality | Falls | NR | Self-reported falls (telephone survey and diary) | Principal component analysis | No /No |
| Wyatt 2020 | United States | 264 F: 162 (61.4%) M: 102 (38.6%) | Median (IQR): 55 (37.7-69.0) | Apple Watch | Heart rate | Abnormal pulse | NR | Electronic health record data of 12-lead ECG, Holter monitor and chest x-ray. | Chi-square tests. | No /No |
| Weiss 2013 | United States | 71 F: 46 (64.8%) M: 25 (35.2%) | 78.3±4.7 | DynaPort accelerometer | Gait Quality | Falls | NR | Self-reported falls | Binary logistic regression | No /No |
| Zhu 2022 | United States | 204 Gender: NR | NR | Samsung Galaxy Watch | Photoplethysmography | Atrial Fibrillation | NR | One-channel ECG (chest patch) | Sample-wise signal quality estimator, hybrid decision model, high-level decision generator, sensor contact monitor | No /No |

ECG: Electrocardiogram.

F: Female.

IQR: Interquartile range.

M: Male.

NR: Not reported.

# Table S5. JBI Critical Appraisal Checklist for Diagnostic Test Accuracy Studies (n=28).

|  | 1.  Sample^1^ | 2.  Design^1^ | 3.  Exclusions^1^ | 4.  Patients | 5.  Index test^1^ | 6.  Threshold^1^ | 7.  Pre-specification | 8. Interpretation | 9.  Target condition^1^ | 10.  Reference result^1^ | 11. Bias | 12. Review question | 13. Time interval^1^ | 14. Receive reference standard | 15. Same reference standard | 16. Analysis^1^ | 17. patient flow | Overall score out of 9^1^ |
| --- | --- | --- | --- | --- | --- | --- | --- | --- | --- | --- | --- | --- | --- | --- | --- | --- | --- | --- |
| Abir 2022 | Unclear | Yes | Unclear | Low | No | Yes | Unclear | Low | Yes | No | Low | Low | Yes | Yes | Yes | No | High | 4 |
| Alavi 2022 | Yes | Yes | Yes | Low | Yes | Yes | Low | Low | Unclear | Unclear | High | Low | Yes | No | Can’t tell | No | High | 6 |
| Caillol 2021 | Unclear | Yes | Yes | Low | Yes | Yes | Low | Low | Yes | Yes | Low | Low | Yes | Yes | Yes | Can’t tell | Unclear | 7 |
| Cleary 2022 | No | Yes | Unclear | Unclear | Unclear | Unclear | Low | Low | Unclear | Unclear | Low | Low | Yes | Yes | Can’t tell | No | Unclear | 2 |
| D’Haese 2021 | Unclear | Yes | Yes | Low | Unclear | Yes | Low | Low | Yes | Unclear | Low | Low | Yes | Yes | Yes | No | High | 5 |
| Gadaleta 2021 | No | Yes | Yes | Low | Yes | Unclear | Low | Low | Yes | Yes | Unclear | Low | Yes | Yes | Yes | No | Low | 6 |
| Guo 2019 | Unclear | Yes | Yes | Low | Unclear | Yes | Low | Low | Yes | Unclear | Low | Low | Yes | Yes | No | No | Low | 5 |
| Hassantabar 2021 | No | Yes | Yes | Low | Unclear | Yes | Low | Low | Yes | Unclear | Low | Low | Yes | Yes | Yes | Can’t tell | Unclear | 5 |
| Hirten 2021 | Yes | Yes | Yes | Low | Unclear | Yes | Low | Low | Yes | Unclear | Low | Low | Yes | Yes | Yes | Yes | Low | 7 |
| Liu 2021 | Unclear | No | Yes | Low |  | Yes | Low | Low | Yes | Unclear | Low | Low | Yes | Yes | Yes |  |  | 4 |
| Lockhart 2021 | Yes | Yes | Yes | Low | Yes | Yes | Low | Low | Yes | Yes | Low | Low | Yes | Yes | Yes | Yes | Low | 9 |
| Lonini 2020 | Unclear | No | Yes | Low | Unclear | Yes | Low | Low | Yes | Unclear | Low | Low | Yes | Yes | Yes | Yes | Low | 5 |
| Lubitz 2022 | Yes | Yes | Yes | Low | Yes | Yes | Low | Low | Yes | Yes | Low | Low | Yes | No | Yes | No | High | 8 |
| Mason 2022 | Yes | Yes | Yes | Low | No | Yes | Unclear | Low | Yes | Yes | Unclear | Low | Yes | No | Can’t tell | Can’t tell | Low | 7 |
| Mishra 2020 | Yes | Unclear | Yes | Low | No | Yes | Unclear | Low | Yes | Yes | High | Low | Yes | Can’t tell | Can’t tell | No | High | 6 |
| Natarajan 2020 | Yes | Yes | Unclear | Low | Yes | Unclear | Low | Low | Yes | Yes | Low | Low | Yes | Yes | Yes | No | Unclear | 6 |
| Nestor 2023 | Unclear | Yes | Yes | Low | Unclear | Unclear | Low | Unclear | Unclear | No | Unclear | Low | Yes | Can’t tell | Can’t tell | Can’t tell | Unclear | 3 |
| Perez 2019 | Yes | Yes | Yes | Low | Unclear | Yes | Low | Low | Yes | Unclear | Low | Low | Yes | Yes | Yes | Yes | Low | 7 |
| Ploux 2021 | Yes | Yes | Yes | Low | Yes | Yes | Low | Low | Yes | Yes | Low | Low | Yes | Yes | Yes | Yes | Low | 9 |
| Quer 2021 | Yes | Yes | Yes | Low | Unclear | Yes | High | Unclear | Unclear | Unclear | High | Low | Yes | No | Can’t tell | No | High | 5 |
| Skibińska 2021 | No | No | Yes | Low | No | Yes | Low | Low | Yes | Unclear | Low | Low | Yes | Yes | Yes | Yes | Low | 5 |
| Skibińska 2022 | Unclear | Unclear | Unclear | Unclear | Unclear | Unclear | Low | Unclear | Unclear | Unclear | Unclear | Low | Yes | Can’t tell | Can’t tell | Can’t tell | Unclear | 1 |
| Smarr 2020 | Yes | Yes | Yes | Low | Unclear | Yes | Low | Low | Yes | Unclear | Low | Low | Yes | Yes | Yes | Yes | Low | 7 |
| Tison 2018 | Unclear | Yes | Yes | Low | Unclear | Yes | Low | Low | Yes | Unclear | Low | Low | Yes | Yes | Yes | Yes | Low | 6 |
| van Schooten 2016 | Unclear | Yes | Yes | Low | No | Yes | Low | Low | Yes | Unclear | Low | Low | Yes | Yes | Yes | Yes | Low | 6 |
| Weiss 2013 | No | Yes | Yes | Low | No | Yes | Low | Low | Yes | Unclear | Low | Low | Yes | Yes | Yes | Yes | Low | 6 |
| Wyatt 2020 | Yes | Yes | Yes | Low | Unclear | Unclear | Unclear | Unclear | Yes | Unclear | Unclear | Low | Yes | Yes | No | Yes | High | 6 |
| Zhu 2022 | Unclear | Yes | Unclear | High | Unclear | Yes | Low | Low | Yes | Unclear | Low | Low | Yes | Yes | Yes | Yes | Low | 5 |
| ^1^Items 1, 2, 3, 5, 6, 9, 10, 13 and 16 contribute to overall score.  Item descriptions: 1. Was a consecutive or random sample of patients enrolled?; 2. Was a case-control design avoided?; 3. Did the study avoid inappropriate exclusions?; 4. Is there concern that the included patients do not match the review question?; 5. Were the index test results interpreted without knowledge of the results of the reference standard?; 6. If a threshold was used, was it pre-specified?; 7. Could the conduct or interpretation of the index test have introduced bias?; 8. Is there concern that the index test, its conduct, or interpretation differ from the review question?; 9. Is the reference standard likely to correctly classify the target condition?; 10. Were the reference standard results interpreted without knowledge of the results of the index test?; 11. Could the reference standard, its conduct, or its interpretation have introduced bias?; 12. Is there concern that the target condition as defined by the reference standard does not match the review question?; 13. Was there an appropriate interval between index test(s) and reference standard?;14. Did all patients receive a reference standard?; 15. Did patients receive the same reference standard?; 16. Were all patients included in the analysis?; 17. Could the patient flow have introduced bias?; | | | | | | | | | | | | | | | | | |  |

# Figure S1. Subgroup analysis of device type for sensitivity of wearable activity trackers for detection of COVID-19.

Note: ‘Various’ refer to studies not using either Fitbit or Oura ring exclusively.

# Figure S2. Subgroup analysis of device type for specificity of wearable activity trackers for detection of COVID-19.

Note: ‘Various’ refer to studies not using either Fitbit or Oura ring exclusively.

# Figure S3. Sensitivity analyses for a) accuracy, b) area-under-curve (AUC) c) sensitivity, and d) specificity of wearable activity trackers for detection of COVID-19, with removal of studies with worst quality assessment.

a) Accuracy

b) Area-under (AUC, %)

c) Sensitivity

d) Specificity

# Figure S4. Sensitivity analyses for a) positive predictive value (PPV), b) sensitivity and c) specificity of wearable activity trackers for detection of atrial fibrillation (AF) and arrythmia (AR), with removal of studies with worst quality assessment.

1. Positive predictive value (PPV)

1. Sensitivity

1. Specificity
